# Smartphone Application for Spastic Ataxias: Cross-Sectional Validation of a Newly Developed Smartphone App for Remote Monitoring in Spastic Ataxias

**DOI:** 10.1007/s12311-025-01820-3

**Published:** 2025-03-24

**Authors:** Ilse H. J. Willemse, Sabato Mellone, Carlo Tacconi, Winfried Ilg, Rebecca Schüle, Matthis Synofzik, Jorik H. Nonnekes, Bart P. C. van de Warrenburg

**Affiliations:** 1 Donders Institute for Brain, Cognition and Behaviour, Department of Neurology, Center of Expertise for Parkinson & Movement Disorders, Radboud University Medical Center, Nijmegen, The Netherlands; 2 Department of Electrical, Electronic and Information Engineering “Guglielmo Marconi”, University of Bologna, Bologna, Italy; 3 mHealth Technologies s.r.l., Bologna, Italy; 4 Section Computational Sensomotorics, Hertie Institute for Clinical Brain Research, Tübingen, Germany; 5 Division of Neurodegenerative Diseases, Department of Neurology, Heidelberg University Hospital and Faculty of Medicine, Heidelberg, Germany; 6 Center for Neurology and Hertie Institute for Clinical Brain Research, University of Tübingen, Tübingen, Germany; 7 Division Translational Genomics of Neurodegenerative Diseases, Hertie-Institute for Clinical Brain Research and Center of Neurology, Tuebingen University Hospital, Hoppe-Seyler-Str. 3, 72076 Tuebingen, Germany; 8 German Center for Neurodegenerative Diseases (DZNE), Otfried-Müller-Str. 23, 72076 Tübingen, Germany; 9 Donders Institute for Brain, Cognition and Behaviour, Department of Rehabilitation, Center of Expertise for Parkinson & Movement Disorders, Radboud University Medical Center, Nijmegen, The Netherlands; 10 Department of Rehabilitation, Sint Maartenskliniek, Nijmegen, The Netherlands

**Keywords:** Spastic ataxia, Smartphone application, App, Digital biomarkers, Remote monitoring

## Abstract

Effective trial-planning in Spastic ataxias (SPAX) is impeded by the absence of validated outcome measures for detecting longitudinal changes. Digital outcome measures show promise, demonstrating sensitivity to disease severity changes in ataxia and strong correlations with clinical scales. The objective of this study was to develop a smartphone application for SPAX (SPAX-app) to obtain valid digital outcome measures for use in clinical trials. The app contains four tasks assessing gait, standing balance (stance), and finger and hand movements. We carried out a validation study in 22 SPAX patients and 10 controls. Subjects performed the tasks three times during one visit. In parallel, we performed Ambulatory Parkinson’s Disease Monitoring (APDM) sensor recordings, Q-motor upper limb assessment, and the Scale for the Assessment and Rating of Ataxia (SARA). Significant correlations were found between the SPAX-app and APDM or Q-motor for step time (*r* = 0.91), all measures of stance (*r* = 0.51–0.87) and duration of hand alternation (*r* = 0.64). No significant correlations were found for variability measures (e.g. standard deviation of step time). All tasks of the SPAX-app, except for stance, can discriminate SPAX from controls and show moderate to high test-retest reliability (ICC 0.67–0.97). Significant correlations were found between SARA and step time (*r* = 0.70), inter-onset interval in finger tapping (*r* = 0.57), and duration of hand alternation (*r* = 0.65). The stance task did not show significant correlations with SARA. With the SPAX-app, we present a set of digital outcome measures for potential use in clinical trials. Longitudinal studies are needed to evaluate whether these measures can track disease progression.

**Supplementary Information:**

The online version contains supplementary material available at 10.1007/s12311-025-01820-3.

## Introduction

Spastic ataxias (SPAX) are a group of rare, genetic neurodegenerative diseases, characterized by progressive spasticity of the lower limbs combined with gait and limb ataxia, dysarthria, and oculomotor disturbances [1]. Even though disease-modifying treatment of SPAX is currently unavailable, recent advancements (e.g. the development of gene therapies) have led to the possibility of genetic and other mechanistic interventions in rare and genetic neurodegenerative diseases, including SPAX [1, 2]. Nevertheless, effective trial-planning in SPAX is impeded by the absence of validated outcome measures capable of detecting longitudinal changes and, subsequently, response to treatment therapies in short time frames [3]. Outcome measures currently used for rating disease severity in SPAX are subjective, rater-dependent, and typically require in-clinic assessments [3]. Moreover, these outcome measures are inadequate to objectively capture patient’s daily life functioning and symptom variability [4, 5].

Digital outcome measures have shown promise in this area, demonstrating sensitivity to small changes in disease severity in people with degenerative cerebellar ataxia and outperforming clinical rating scales [6, 7]. Multiple studies utilizing wearable body-worn sensors in SPAX [5, 8] or ataxia [9–12] within lab-based settings have demonstrated that these outcome measures correlate well with disease severity, can discriminate patients from healthy controls, and capture disease progression. Furthermore, several studies have demonstrated that digital outcome measures, captured by three body-worn inertial sensors, can be reliably assessed in real-life, with a significant correlation to disease severity [7, 13]. As a result, this approach is recommended for future clinical trials [14]. However, the technologies used within these studies are costly and still inadequate for longer monitoring periods at home in larger patient groups. The choice of the system will depend on the study aim. For longer monitoring periods at home, alternative digital technologies such as built-in wearable sensors in smartphone and tablet applications (apps) may offer a more practical solution, as they are able to actively and passively monitor symptoms in daily life [15].

To date, only five apps for ataxias have been developed that are supported by adequate research [16–22]. However, these apps can only capture a specific symptom domain of ataxia [17–20], such as stance, gait or fine motor skills, or capture videos rather than utilizing sensor signals [16].

The aim of this study was to develop and validate a new digital outcome measure tool for use in clinical trials in SPAX. We developed a smartphone application, named SPAX-app, for quantitative assessments of gait, stance, upper limb functioning, and speech, complemented by a single question that asks for the patients’ global impression of disease severity. This current study only focuses on the four motor assessments of the SPAX-app (gait, stance and two tasks assessing upper limb functioning). We here describe the development as well as the clinical and technological cross-validation processes of the SPAX-app. Furthermore, the present study explored the feasibility of the app under real-life conditions at home, including the collection of user feedback.

## Method

### SPAX-App

**Fig. 1:**
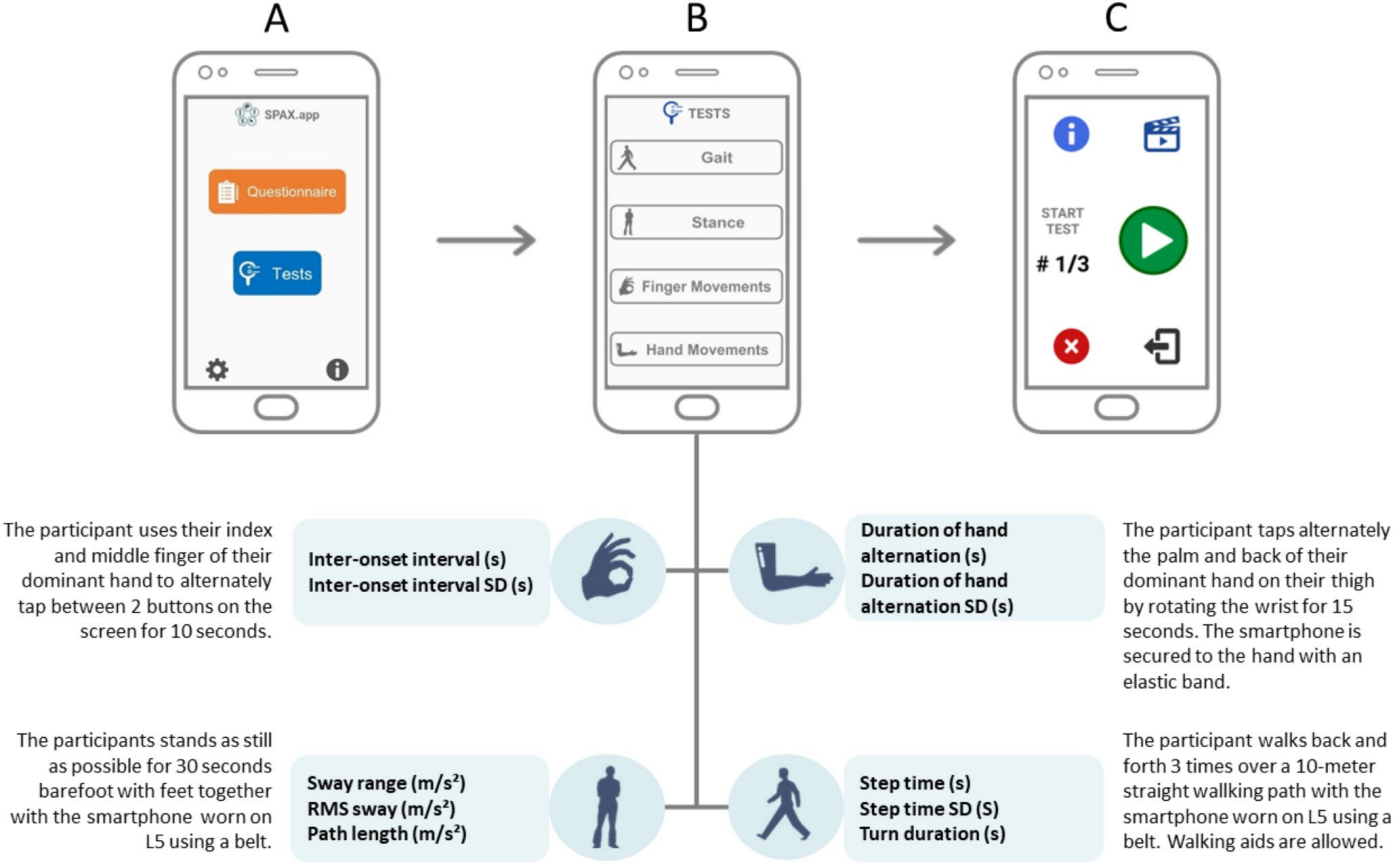
The home screen of the SPAX-app contains two buttons (**A**). The top button, the “Questionnaire”, asks users to rate the severity of their symptoms at the time of use on a scale from 0 to 10. The “Tests” button leads the user to 4 short motor tasks (**B**) assessing gait, stance, and finger and hand movements. Tests can only be assessed after completing the question. Users can then start a task by pressing on the green play button (**C**). Each task must be repeated three times in a row to complete one test session

This study was part of the PROSPAX study, a prospective, international, longitudinal, multicenter, natural progression study in spastic ataxias (ClinicalTrials.gov, No: NCT04297891). The SPAX-app (Fig. 1) is designed for patients with SPAX and is able to measure patients’ capacity by remote, quantitative assessments of gait, stance and upper limb functioning and to obtain the patients’ perception of health status by asking a question regarding symptom severity. Written and video instructions for each task are available in the app along with vocal instructions while performing the tasks. The SPAX-app, including written and video instructions of the tasks, was available in Dutch. Completing all tasks takes about 30 min. Patients were recruited from the Radboud University Medical Center in Nijmegen the Netherlands.

### Data Collection

#### Lab-Based Validation Study

We first carried out a technical and clinical validation study in 22 SPAX patients and 10 healthy controls in a lab-based environment, which will be referred to as the validation cohort.

After rating the severity of their symptoms in the app, subjects were asked to perform all or some of the tasks of the app based on their capabilities. A subset of the subjects (5 SPG7, 1 ARSACS, 5 healthy controls) was asked to perform the stance task with feet in neutral position using a reference board in addition to the instructed stance task (Fig. 1) to observe if the same task performed with feet together could better discriminate between SPAX and healthy controls. Subjects performed the prescribed tasks of the app three times in a row during one in-clinic visit under supervision of a researcher (IW), along with similar tasks with already validated digital assessment tools to evaluate the apps’ technological concurrent validity. Subjects were fitted with three inertial sensors (Opals by APDM Wearable Technology-an ERT company, Portland, OR, USA) while performing the gait and stance tasks. One sensor was placed on the dorsum of each foot and one sensor was placed at the lumbar spine at L5. In addition to the SPAX-app tasks, subjects performed two tasks for cross-validation: a finger tapping task and fast alternating hand movements task on the Q-motor system (QuantiMedis GmbH, Münster, Germany) [23]. The Q-motor finger tapping was performed by tapping as quickly as possible for 20 s with the index finger of the dominant hand on a force transducer. The Q-motor fast alternating hand movements task was performed by tapping alternating and as quickly as possible for 20 s with the palm and back of the hand on a force transducer. All tasks were performed in a fixed order during the in-clinic visit to ensure consistency across all subjects. In exceptional cases the order of the tests was adjusted for logistical reasons.Moreover, we conducted frequently used clinical scales and instruments to evaluate the apps’ clinical construct validity: the SARA, Friedreich’s Ataxia Rating Scale – Activities of Daily living (FARS-ADL) and Nine Hole Peg Test (9HPT) were assessed by a trained examiner [24–26].

#### Home-Based Pilot Study

We next conducted a pilot study to assess for a learning effect of the tasks and explore the feasibility of using the app at home. We included a subset of the validation cohort and one additional subject. This pilot cohort consisted out of 17 SPAX patients (11 SPG7 and 6 ARSACS).

Subjects performed the four tasks in their homes for 4 weeks after receiving one in-clinic training session. Subjects decided, in consultation with the researcher, which tasks they could perform at home based on their capabilities. They completed the tasks two times a week, on one weekday and one weekend day of their choosing, and were asked to perform the tasks on the same days of the week and the same part of the day for the whole period. All participants completed the system usability scale (SUS) [27] after 4 weeks.

### Data Analysis

For both the validation and pilot study, we selected at least two and a maximum of three outcome measures per task (Fig. 1*)*. For the gait task we selected step time, the standard deviation (SD) of step time and turn duration. For the stance task we selected sway range, the root mean square (RMS) of sway and path length. For the finger movements task we selected inter-onset interval and its SD. For the hand movements task we selected duration of hand alternation and duration and its SD. For the gait and stance task of the SPAX-app only the smartphone sensor positioned at L5 is used. Therefore, we selected a set of speed-related measures that are known to be reliably captured using only one sensor at this location [11, 12, 28]. For the finger and hand movements task we selected a set of speed-related measures as well as previous research showed that speed (captured in frequency and inter-peak-interval) and its variability measured during quantitative motor assessment of upper limb ataxia correlated strongly with ataxia severity measures [23]. A detailed description of the data analysis can be found in Supplemental information 1. Inorder to investigate the feasibility of the SPAX-app, we calculated the compliance during the 4 weeks. The compliance was defined as the number of days the SPAX-app was used according to the correct procedure, expressed as a percentage of the predetermined number of days.

### Statistical Analysis

Statistical analysis was performed using MATLAB version 2023B. Normality testing showed that only the data from healthy controls in the validation study were not normally distributed. To assess concurrent validity, a first Spearman correlation analysis was performed in the validation cohort between the gait and stance measures extracted by the SPAX-app (Fig. 1) and those obtained with APDM’s mobility lab software using Spearman’s rho ($\:{\text{r}}_{\text{s}})$. A similar analysis was performed for the finger and hand movement measures extracted by the SPAX-app (Fig. 1), comparing them with the corresponding outcome measures from the Q-motor system. A test-retest reliability analysis was carried out on each of the outcome measures extracted by the SPAX-app by calculating the intraclass correlation coefficient (ICC) between the first and third consecutively performed tasks of the app. Subsequently, a Mann-Whitney U test was carried out between the two groups in order to assess discriminative validity of the outcome measures. In addition, a Mann-Whitney U test was carried out to determine if there was a significant difference in age and gender between the two groups. The Bonferroni correction method was employed to address the issue of multiple comparisons in the between-group analyses for each task of the SPAX-app. To assess construct validity, a second Spearman correlation analysis was performed to examine the relationship between the outcome measures of the SPAX-app and the clinical outcome measures. For gait and stance tasks, clinical outcome measures include the SARA, SARA posture and gait subscore (SARAp&g), FARS-ADL and SPAX-app question, while for finger and hand movements, they include the SARA, SARA upper limb subscore (SARAul), FARS-ADL, 9HPT, and SPAX-app question. The SARAul is composed of items 5, 6, and 7 of the SARA. We repeated all validation analyses for the gait task in a subgroup, upon excluding patients who used walking aids [14]. To test for a learning effect in the pilot cohort, a paired t-test was performed on the second and last testing day of the 8-week period at home. In order to assess the consistency of the performed tasks of the app in patients with SPAX in different environments, we evaluated the correlation between the results obtained in the validation cohort in a lab-based setting and at home using Spearman’s rho.

## Results

### Lab-Based Validation Study

All subjects of the validation cohort (17 SPG7, 5 ARSACS, and 10 healthy controls) performed the finger and hand movements tasks of the app. Among the SPAX patients, 36% were male with an average age of 53 years (range:27–72). Among the healthy controls, 50% was male with an average age of 46 years (range:19–71). The SPAX population had an average score of 14.2 on the Scale for Assessment and Rating of Ataxia (SARA) (range:2–29). A number of 16 subjects with SPAX (15 SPG7, 1 ARSACS) and 10 healthy subjects also performed the gait and stance tasks of the app. The remaining 6 subjects were wheelchair-bound and not able to walk without support of another person. Two subjects with SPAX used a walker while performing the gait task and one subject used one stick. Five participants adjusted their stance or reached for support to maintain balance during a single trial of the stance task. A subset of the subjects in the validation cohort (15 SPG7, 5 ARSACS and 5 healthy controls) also completed the finger tapping and hand turning task using the Q-motor system. SARA scores per task for the subjects with SPAX are presented in Supplemental Fig. 1, and characteristics of the subjects with SPAX for each task are provided in Supplemental Table 1.

#### Gait

**Fig. 2:**
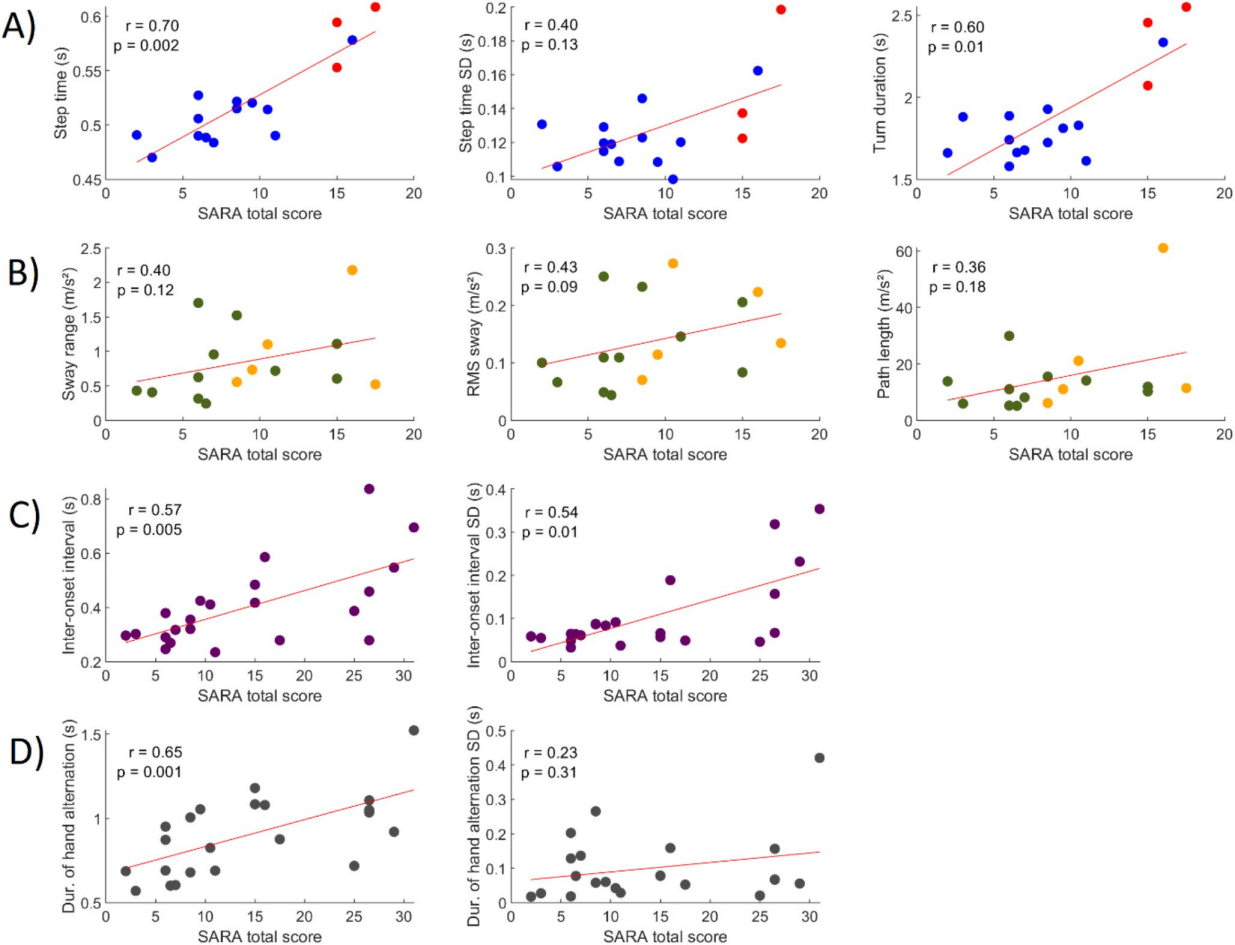
Scatterplots illustrating the relationship between between the SARA total score and the outcome measures of the gait (**row A**; red dots are patients who used a walking aid during the task), stance (**row B;** orange dots are patients who adjusted their stance or reached for support during the task), finger movements (**row C**) and hand movements (**row D**) tasks of the SPAX-app. Each plot displays the Spearman correlation coefficient (r) and corresponding p-value

There was a very strong significant correlation between the SPAX-app and APDM sensors for the step time ($\:{\text{r}}_{\text{s}}$=0.91); however, no significant correlation was found for the standard deviation of the step time ($\:{\text{r}}_{\text{s}}$=0.33) and turn duration ($\:{\text{r}}_{\text{s}}$=-0.01) (Table 1). Furthermore, all gait outcome measures demonstrated high test-retest reliability with an ICC ≥ 0.89 (Table 2). A significant difference (*P* < 0.01, Supplemental Table 2) was found between subjects with SPAX and healthy subjects for the gait outcome measures, but not for age and gender. Further analysis revealed a strong significant correlation with the SARA and SARAp&g scores for the step time ($\:{\text{r}}_{\text{s}}$=0.70 and $\:{\text{r}}_{\text{s}}$=0.81) and a moderate correlation with SARA and SARAp&g for the turn duration ($\:{\text{r}}_{\text{s}}$=0.60 and $\:{\text{r}}_{\text{s}}$=0.53). The standard deviation of the step time was only significantly correlated to the SARAp&g ($\:{\text{r}}_{\text{s}}$=0.50), but not to the total SARA ($\:{\text{r}}_{\text{s}}$=0.40) (Table 2; Fig. 2). The symptom severity question in the app demonstrated a strong and statistically significant correlation with all gait outcome measures ($\:{\text{r}}_{\text{s}}$≥0.66) (Table 2). For the FARS-ADL, there was a significant correlation with the mean step time ($\:{\text{r}}_{\text{s}}$=0.80) and the turn duration ($\:{\text{r}}_{\text{s}}$=0.79).

**Table 1:** Cross-validation of the four short motor tasks with APDM and Q-motor outcome measures (Spearman correlation) in subjects with SPAX

Outcome measure	SPAX-app	System for cross-validation APDM wearable technologies/Q-motor system	Spearman correlation
**Gait (** ***n*** ** = 16)**			
Step time (s)	0.52 ± 0.04	0.59 ± 0.13	0.91**
Step time SD (s)	0.13 ± 0.02	0.03 ± 0.04	0.33
Turn duration (s)	1.90 ± 0.30	2.21 ± 0.54	-0.01
**Stance (** ***n*** ** = 16)**			
Sway range (m/s²)	0.86 ± 0.54	1.05 ± 0.46	0.51*
RMS sway (m/s²)	0.14 ± 0.08	0.13 ± 0.06	0.75**
Path length (m/s²)	15.09 ± 13.82	13.21 ± 8.82	0.87**
**Finger movements (** ***n*** ** = 16)**			
Inter-onset interval (s)	0.41 ± 0.18	0.32 ± 0.14	0.49
Inter-onset interval SD (s)	0.11 ± 0.10	0.03 ± 0.02	0.21
**Hand movements (** ***n*** ** = 16)**			
Duration of hand alternation (s)	0.91 ± 0.25	0.57 ± 0.18	0.64**
Duration of hand alternation SD (s)	0.10 ± 0.09	0.12 ± 0.07	0.12

* *p* ≤ 0.05; ** *p* ≤ 0.01

SPAX = spastic ataxias; SD = Standard deviation; RMS = Root mean square

**Table 2:** Cross-sectional validation of the four short motor of the SPAX-app with clinical measures in subjects with SPAX

Outcome measure	SARA Spearman correlation	SARAp&g Spearman correlation	SARAul Spearman correlation	SPAX-app Question Spearman correlation	ICC (95% CI)
**Gait (** ***n*** ** = 16)**					
Step time (s)	0.70**	0.81**	-	0.74**	0.89** **(0.69–0.96)**
Step time SD (s)	0.40	0.50*	-	0.66**	0.90** **(0.73–0.97)**
Turn duration (s)	0.60*	0.53*	-	0.66**	0.95** **(0.87–0.98)**
**Stance (** ***n*** ** = 16)**					
Sway range (m/s²)	0.40	0.39	-	0.03	0.51 (-0.39-0.83)
RMS Sway (m/s²)	0.43	0.50*	-	-0.07	0.43 (-0.63-0.80)
Path length (m/s²)	0.36	0.44	-	-0.01	0.84** **(0.56–0.95)**
**Finger movements (** ***n*** ** = 22)**					
Inter-onset interval (s)	0.57**	-	0.48*	0.10	0.97** **(0.92–0.99)**
Inter-onset interval SD (s)	0.54**	-	0.44*	-0.13	0.79** **(0.49–0.91)**
**Hand movements (** ***n*** ** = 22)**					
Duration of hand alternation (s)	0.65**	-	0.64**	0.58**	0.97** **(0.92–0.98)**
Duration of hand alternation (s)	0.23	-	0.37	0.32	0.67** **(0.20–0.86)**

* *p* ≤ 0.05; ** *p* ≤ 0.01

SPAX = spastic ataxias; SARA = Scale for Assessment and Rating of Ataxia; SARAp&g = SARA posture and gait score; SARAul = SARA upper limp score; SD = Standard deviation; RMS = Root mean square; CI = Confidence

The measures of patients that used a walking aid fitted the expected trajectories (Fig. 2A). In addition, when validating the gait task upon exclusion of their data, we observed comparable results in terms of concurrent and discriminative validity. However, correlations between SARA total score and gait parameters decreased and no significant correlations remained, except for the SARAp&g score and step time (Supplemental Tables 4–6).

#### Stance

No significant differences (*p* > 0.01; Supplemental Table 3) were found between healthy individuals and patients in any of the outcome measures of the stance task when performed with feet in neutral position. As a result, the task was modified to a feet-together stance. The results presented here reflect the outcomes of this adjusted task. There was a moderate to strong significant correlation between the SPAX-app and APDM for the sway range ($\:{\text{r}}_{\text{s}}$=0.51), RMS of the sway ($\:{\text{r}}_{\text{s}}$=0.75) and path length ($\:{\text{r}}_{\text{s}}$=0.87) (Table 1). Further statistical tests revealed a moderate to good test-retest reliability with an ICC between 0.43 and 0.84 for the stance outcome measures (Table 2). We only found a significant difference (*P* < 0.01, Supplemental Table 2) for the path length between the two groups and no significant difference was found for age and gender. Further analysis revealed no statistically significant correlations with the SARA and SARAp&g for the sway range ($\:{\text{r}}_{\text{s}}$=0.40 and $\:{\text{r}}_{\text{s}}$=0.39) and path length ($\:{\text{r}}_{\text{s}}$=0.36 and $\:{\text{r}}_{\text{s}}$=0.44). There was a significant correlation with the SARAp&g, but not with SARA, for the RMS of sway ($\:{\text{r}}_{\text{s}}$=0.50 and $\:{\text{r}}_{\text{s}}$=0.43) (Table 2; Fig. 2). The symptom rating question in the app demonstrated no statistically significant correlation with any of the outcome measures of the stance task (Table 2). In addition, we found no significant correlation with the FARS-ADL for any of the stance outcome measures.

#### Finger Movements

There was no significant correlation between the SPAX-app and the Q-motor system for the inter-onset interval ($\:{\text{r}}_{\text{s}}$=0.49) and standard deviation of the inter-onset interval ($\:{\text{r}}_{\text{s}}$=0.21) (Table 1). Further statistical tests revealed a high test-retest reliability with an ICC ≥ 0.79 for both outcome measures of the finger movement task (Table 2). A significant difference (*P* < 0.01, Supplemental Table 2) was found between the two groups for the inter-onset interval of the finger movements task of the app but not for the standard deviation of the inter-onset interval, age and gender. Further analysis revealed a significant moderate to strong correlation with the SARA and SARAul for the inter-onset interval ($\:{\text{r}}_{\text{s}}$=0.57 and $\:{\text{r}}_{\text{s}}$=0.48) and the standard deviation of the inter-onset interval ($\:{\text{r}}_{\text{s}}$=0.54 and $\:{\text{r}}_{\text{s}}$=0.44) (Table 2; Fig. 2). The symptom rating question in the app demonstrated no statistically significant correlation with both of the outcome measures of the finger movements task (Table 2). We did observe a significant correlation with the nine-hole pegboard test for the inter-onset interval ($\:{\text{r}}_{\text{s}}$=0.62) and the standard deviation of the inter-onset interval ($\:{\text{r}}_{\text{s}}$=0.61). For the FARS-ADL, there was only a significant correlation with the mean inter-onset interval ($\:{\text{r}}_{\text{s}}$=0.53).

#### Hand Movements

There was a significant strong correlation between the SPAX-app and the Q-motor system for the duration of hand alternation ($\:{\text{r}}_{\text{s}}$=0.64). However, no significant correlation was found for the standard deviation of the duration of hand alternation ($\:{\text{r}}_{\text{s}}$=0.12) (Table 1). Further statistical tests revealed a high test-retest reliability for the duration of hand alternation with an ICC of 0.97 and an ICC of 0.67 for the standard deviation of the turn duration (Table 2). A significant difference (*P* < 0.05, Supplemental Table 2) was found between the two groups for the duration of hand alternation only and no significant difference was found for age and gender. Further analysis revealed a significant strong correlation with the SARA and SARAul for duration of hand alternation ($\:{\text{r}}_{\text{s}}$=0.65 and $\:{\text{r}}_{\text{s}}$=0.64), but no significant correlation was found for the standard deviation of the duration of hand alternation ($\:{\text{r}}_{\text{s}}$=0.23 and $\:{\text{r}}_{\text{s}}$=0.37) (Table 2; Fig. 2). The symptom rating question in the app demonstrated only a significant correlation with the duration of hand alternation ($\:{\text{r}}_{\text{s}}$=0.58) (Table 2). In addition, we found no significant correlation with the nine-hole pegboard test and the FARS-ADL for any of the hand movements outcome measures.

### Home-Based Pilot Study

A number of 17 subjects with SPAX (11 SPG7, 6 ARSACS) performed the tasks in the SPAX-app at home twice a week for four weeks. Among the SPAX patients, 47% were male with an average age of 53 years (range:23–72) and the average SARA score was 15.9 (range:6–29). The stance task was executed with feet in neutral position (with the use of a reference board) instead of feet together during the home-based pilot study. Given the absence of discernible differences between SPAX and healthy controls in the stance task with feet in neutral position during the lab-based validation (Supplemental Table 3), we decided to not include this task in the analyses of the pilot study.

#### Feasibility

Subjects with SPAX completed 89.7% of the predetermined test days (Supplemental Fig. 2). They did not use the app for 4.4% and did not complete all tests for 3.7% of the days. Subjects had the opportunity to complete the tests one day later if the predetermined test day was missed. This was done three times (2.2%).

Of the 17 subjects, 15 completed the SUS. The SPAX-app received an average SUS score of 65 (range: 0-100, with scores ranging from 52.5 to 97.5) from SPAX subjects, with a standard deviation of 8.4, indicating a moderate to good level of usability. A majority of the SPAX subjects (87%) found the SPAX-app easy to use. Opinions differed as to whether subjects wanted to use the SPAX-app frequently. Just under half of those who answered this questionnaire reported “Neutral”, while 40% would like to use the app frequently, and 13% would not.

#### Learning Effect

No significant differences were observed over the 4-week period in relation to the hand movements task (Supplemental Table 7). However, SPAX patients presented a significant decrease in the inter-onset interval measured during the finger movements task. Additionally, in the gait task, a significant decrease was presented in the standard deviation of step time. No significant difference was found for the other outcome measures of the gait task.

#### Lab Vs. Home Correlation

We observed a significant correlation between most of the outcome measures of the SPAX-app when performed at home and in a lab-based setting in (Supplemental Table 8). However, the standard deviation of the step time (gait task) and the standard deviation of the duration of hand alternation (hand movements task) did not demonstrate significant correlations between the two settings.

## Discussion

This study presents a lab-based validation and home-based pilot study of four short motor tasks within the SPAX-app. The results show moderate to strong cross-correlations for the SPAX-app with APDM wearable sensors and the Q-motor system (concurrent validity) for mean measures of the SPAX-app, but not for measures of variability. Moreover, all measures can discriminate SPAX patients from healthy controls (discriminative validity) and show a high test-retest reliability except for the sway range, RMS of sway and duration of hand alternation. In addition, we found that most SPAX-app measures show moderate to good correlations with ataxia severity measures (construct validity) except for those of the stance task. Taking these validation criteria into account, we identified four measures (step time, inter-onset interval and standard deviation of inter-onset interval in finger tapping, and the duration of hand alternation) taken from the gait, finger movements, and hand movements task of the app that can be considered for use in clinical trials. It should be acknowledged, however, that only the mean step time and inter-onset interval in finger tapping, out of these four measures, showed a significant correlation with daily activities measured by the FARS-ADL.

An unanticipated result was that only the stance measure ‘path length’ was both discriminative between groups and highly reliable in test-retest, but it did not correlate with SARA or SARAp&g. This suggests the apps’ current stance task is unsuitable for clinical SPAX trials. Furthermore, we did not find strong significant correlations with clinical measures of ataxia severity (e.g. SARA) both in the stance outcome measures of the app and of APDM (Supplemental Table 9). These findings are surprising, as other studies using APDM wearable sensors found strong correlations with SARA in patients with SCA and Friedreich’s ataxia performing the same task [11, 12]. Two factors likely explain these differences. First, our sample size was smaller, which might have affected the statistical power. Second, our cohort had a limited number of participants in the early-disease stages and none in the pre-ataxic stages, where postural sway measures have shown to be strongly related to ataxia severity [29–31].

In terms of concurrent validation, we found that mean measures of the SPAX-app had better cross-correlations with APDM and the Q-motor data than measures of variability (Table 1). The poor correlation for the standard deviation of step time ($\:{\text{r}}_{\text{s}}$=0.33) may be due to APDM’s mobility lab software using foot sensor data, while we used data from the SPAX-app sensor placed on L5. Furthermore, we found an unexpectedly low correlation for the turn duration of the gait task ($\:{\text{r}}_{\text{s}}$=-0.01), which may have been caused by limitations of APDM’s mobility lab software in the detection of turns. Visual inspection of the raw gait data showed that the software often detects multiple turns during a single turn in patients with severe walking disabilities [32]. This result is difficult to explain, but might relate to a compensatory turning strategy previously observed in ataxic patients [33, 34]. Ataxic patients tend to use a strategy focused on using more steps during a turn than the so-called ‘spin’ during a turn, and they reduce and increase the body’s speed more slowly during 180° turns compared to healthy controls [33, 34]. The apps’ finger and hand movement tasks showed lower cross-correlation with Q-motor output compared to gait and stance tasks with APDM. This is likely due to differences in how these tasks are performed on the Q-motor system in comparison to the SPAX-app.

In terms of construct validity, we found that most outcome measures of the app show moderate to good correlations with ataxia severity measures except those of the stance task, the step time SD and the duration of hand alternation SD. It is striking that variability in step time does not show a significant correlation with SARA, while previous studies indicated that measures of spatiotemporal variability in gait correlate well with ataxia severity [6, 9, 10, 29, 30]. We therefore checked the correlations between variability in step time calculated by APDM and SARA in our group of SPAX patients. Here, we did find a significant correlation between the variability in step time and SARA ($\:{\text{r}}_{\text{s}}$=0.76, Supplemental Table 9) which was not found with the SPAX-app. It is noteworthy that the correlations between SARA and mean step time are comparable for the SPAX-app and APDM sensors. This implies limitations in the SPAX-app to capture certain aspects of abnormal motor performance that are, based on prior research, relevant, specific, and sensitive to change in this group [6, 10, 35].

This current study focuses on gait, balance and upper limb functioning assessed by the SPAX-app, which also includes a speech task as mentioned in the introduction. Incorporating additional assessments, such as speech or oculomotor function, in future smartphone applications for ataxias could be valuable [21]. Some patients, as shown in Supplemental Fig. 1, are unable to participate in all tasks, highlighting the potential benefit of broadening the scope of the app. Incorporating these assessments could enhance the apps’ utility in both research and clinical practice across the full spectrum of ataxia.

This study has some limitations. First, we primarily selected speed-dependent outcome measures that assess the capacity of patients instead of ataxia-specific measures. This choice was driven by the limitations of smartphone-based assessments, which rely on a single sensor. Consequently, our results are less comparable to previous studies that utilized multiple sensors approaches focused on ataxia-specific outcomes. Second, the small sample size in our study may have reduced statistical power, potentially causing some outcome measures not reaching statistical significance for clinical correlations. Particularly the small number of healthy controls in our study may have reduced the statistical power, leading to lower significance values for the group differences. This limitation could have resulted in missing potential outcome measures that might have revealed significant differences between the two groups if a larger sample of healthy controls had been included. Finally, the study did not evaluate whether outcome measures of the SPAX-app can track disease progression. The findings of the pilot study suggest a potential learning effect over a four-week period for the finger movements and gait tasks. Further research with larger longitudinal datasets is required to assess the learning effect over extended periods and to evaluate sensitivity to longitudinal change of the four best performing outcome measures of the app.

## Conclusion

The results of this research support the idea that we can use smartphone applications to monitor people with (spastic) ataxias at home. With the SPAX-app, we present a set of digital outcome measures, including step time, inter-onset interval in finger tapping, standard deviation of inter-onset interval in finger tapping, and the duration of hand alternation for potential use in clinical trials. Nevertheless, longitudinal studies are needed to evaluate whether these measures can track disease progression.

## Electronic Supplementary Material

Below is the link to the electronic supplementary material.

Supplementary Material 1

## Data Availability

The data that support the findings of this study are available upon reasonable request.
