# Supplementary material for: Smartphone Application for Spastic Ataxias: Cross-Sectional Validation of a Newly Developed Smartphone App for Remote Monitoring in Spastic Ataxias
Source: Cerebellum. 2025 Mar 24;24(3):71. doi: 10.1007/s12311-025-01820-3 (PMC11933166; doi:10.1007/s12311-025-01820-3)
Supplement: Supplementary file 1 — Supplementary Material 1 [file 12311_2025_1820_MOESM1_ESM.docx]

**Supplemental Information 1 –** Description of data analysis for the validation and pilot study.

For the validation study, we selected at least two and a maximum of three outcome measures per task of the app. For the gait task we calculated the step time, standard deviation of the step time and the turn duration. For the stance task we calculated the sway range, root mean square (RMS) of the sway range and the path length. For the finger movements task we calculated the inter-onset interval and standard deviation of the inter-onset interval. For the hand movements task we calculated the duration of hand alternation and the standard deviation of the duration of hand alternation. Calculation of all outcome measures was carried out using MATLAB version 2023B. While performing the gait, stance and hand movements tasks in the SPAX-app, the app stores the raw data from the accelerometer and gyroscope of the smartphone. It also stores the raw data from the touchscreen presses during the finger movements task. The raw acceleration data of the smartphone sensor was filtered using a 20 Hz lowpass zero phase-shift Butterworth filter for analysis of the gait task. The data was processed using continuous wavelet transform, and the number of steps in a 10-meter segment were determined using MATLAB’s findpeaks function after detection of straight walking segments [1, 2]. The mean duration of a single step was determined by calculating the average time required for one step, and the corresponding standard deviation was also computed. The step time, standard deviation of the step time and turn duration measured with the inertial sensors were obtained by APDM’s mobility lab software. For analysis of the stance task we used the method described in earlier research to calculate the sway range, RMS of the sway and the path length[3-5]. The sway range, RMS of sway and path length measured with the inertial sensors were obtained by APDM’s mobility lab software. The app recorded the time points when a button is tapped during the finger movements task. The inter-onset interval was determined as the time between a tap of the index finger and a tap of the middle finger. The mean and standard deviation of the inter-onset interval was calculated for each trial and for the three consecutive trials combined. The raw gyroscope data of the smartphone was integrated and filtered using a 0.08 Hz lowpass filter for analysis of the hand movements task. The duration of hand alternation is the time of one successive turn of the hand (pronation or supination). The mean and standard deviation of the duration of hand alternation was calculated for each trial and for the three consecutive trials combined. The team at QuantiMedis analyzed the raw data from the finger tapping and fast alternating hand movement tasks performed on the Q-motor device, calculating the tapping frequency, mean inter-onset interval, and standard deviation. They then provided us with the average values for each outcome measure for each participant. For all four tasks of the app, the average per trial and the average over the three consecutive trials was calculated.

In the pilot study, we computed the same outcome measures from the SPAX-app as described in the section above. In order to investigate the feasibility of the SPAX-app, we calculated the compliance during the 4 weeks. The compliance was defined as the number of days the SPAX-app was used according to the correct procedure, expressed as a percentage of the predetermined number of days.

**Supplemental Fig. 1** - The SARA total scores for the SPAX subjects who completed the four short motor tasks of the SPAX-app are displayed for each task. The red asterisk represents the average SARA total score for that specific task.

**Supplemental Table 1:** Characteristics of the subgroups of SPAX patients in het validation study.

|  | **Gait**  **(SPAX-app)***  **N = 16** | **Stance**  **(SPAX-app)***  **N = 16** | **Finger movements (SPAX-app)**  **N = 22** | **Hand movements (SPAX-app)**  **N = 22** | **Q-motor**  **N = 16** |
| --- | --- | --- | --- | --- | --- |
| **SPG7/ARSACS** | 15/1 | 15/1 | 17/5 | 17/5 | 11/5 |
| **Age (years)** | 52.88 ± 10.59 | 52.88 ± 10.59 | 53.41 ± 10.76 | 53.41 ± 10.76 | 54.38 ± 11.14 |
| **Male %** | 44% | 44% | 36% | 36% | 44% |
| **Age of onset (years)** | 38.38 ± 14.03 | 38.38 ± 14.03 | 33.69 ± 16.87 | 33.69 ± 16.87 | 31.51 ± 18.37 |
| **SARA total score** | 9.25 ± 4.63 | 9.25 ± 4.63 | 14.20 ± 9.22 | 14.20 ± 9.22 | 16.06 ± 8.88 |

*Gait and stance tasks were performed while wearing APDM sensors.

**Supplemental Table 2:** Group differences (p-value) for the outcome measures of the four SPAX-app task, age and gender.

| **Outcome measure** |  |  | **p-value***  Mann-Whitney U |
| --- | --- | --- | --- |
| **Gait** | **HC (n=10)** | **SPAX (n=16)** |  |
| Age (years) | 46.80 ± 15.03 | 52.88 ± 10.59 | 0.45 |
| Male (%) | 50% | 44% | 0.78 |
| Step time (s) | 0.45 ± 0.02 | 0.52 ± 0.04 | **0.00004** |
| Step time SD (s) | 0.09 ± 0.008 | 0.13 ± 0.02 | **0.00009** |
| Turn duration (s) | 1.50 ± 0.09 | 1.90 ± 0.30 | **0.00004** |
|  |  |  |  |
| **Stance** | **HC (n=10)** | **SPAX (n=16)** |  |
| Age (years) | 46.80 ± 15.03 | 52.88 ± 10.59 | 0.45 |
| Male (%) | 50% | 44% | 0.78 |
| Sway range (m/s²) | 0.48 ± 0.27 | 0.86 ± 0.54 | 0.03 |
| RMS sway (m/s²) | 0.07 ± 0.02 | 0.14 ± 0.08 | 0.02 |
| Path length (m/s²) | 6.17 ± 1.42 | 15.09 ± 13.82 | **0.007** |
|  |  |  |  |
| **Finger movements** | **HC (n=10)** | **SPAX (n=22)** |  |
| Age (years) | 46.80 ± 15.03 | 53.41 ± 10.76 | 0.45 |
| Male (%) | 50% | 36% | 0.49 |
| Inter-onset interval (s) | 0.24 ± 0.09 | 0.40 ± 0.15 | **0.003** |
| Inter-onset interval SD (s) | 0.05 ± 0.007 | 0.11 ± 0.09 | **0.007** |
|  |  |  |  |
| **Hand movements** | **HC (n=10)** | **SPAX (n=22)** |  |
| Age (years) | 46.80 ± 15.03 | 53.41 ± 10.76 | 0.45 |
| Male (%) | 50% | 36% | 0.49 |
| Duration of hand alternation (s) | 0.64 ± 0.06 | 0.90 ± 0.23 | **0.003** |
| Duration of hand alternation SD (s) | 0.05 ± 0.05 | 0.10 ± 0.10 | 0.08 |
|  |  |  |  |

**p-values were Bonferroni corrected. A p-value of ≤ 0.010 was considered statistically significant for the gait and stance task. A p-value of ≤ 0.0125 was considered statistically significant for the finger and hand movements task.
SPAX = spastic ataxias; HC = healthy controls; SD = Standard deviation; RMS = Root mean square.*

**Supplemental Table 3:** Group differences (p-value) for the stance task of SPAX-app with feet in neutral position.

| **Stance** | **HC (n=5)** | **SPAX (n=7)** | **p-value***  Mann-Whitney U |
| --- | --- | --- | --- |
| Age (years) | 43.20 ± 13.41 | 49.71 ± 12.78 | 0.22 |
| Male (%) | 40% | 86% | 0.30 |
| Sway range (m/s²) | 0.33 ± 0.15 | 0.35 ± 0.16 | 0.76 |
| RMS sway (m/s²) | 0.05 ± 0.02 | 0.06 ± 0.02 | 0.64 |
| Path length (m/s²) | 4.88 ± 2.28 | 5.45 ± 2.63 | 0.53 |
|  |  |  |  |

**p-values were Bonferroni corrected. A p-value of ≤ 0.010 was considered statistically significant.
SPAX = spastic ataxias; HC = healthy controls; RMS = Root mean square.*

**Supplemental Table 4:** Cross-validation of the gait tasks of the SPAX-app with APDM (Spearman correlation) in subjects with SPAX excluding the use of walking aids.

| **Outcome measure** | **SPAX-app** | **System for cross-validation APDM wearable technologies** | **Spearman correlation** |
| --- | --- | --- | --- |
| **Gait (n=13)** |  |  |  |
| Step time (s) | 0.51 ± 0.03 | 0.54 ± 0.05 | **0.87**** |
| Step time SD (s) | 0.12 ± 0.02 | 0.02 ± 0.01 | 0.10 |
| Turn duration (s) | 1.80 ± 0.20 | 2.19 ± 0.50 | -0.08 |

** p ≤ 0.05; ** p ≤ 0.01
SPAX = spastic ataxias; SD = Standard deviation.*

**Supplemental Table 5:** Group differences (P-value) and test-retest reliability (ICC) for the outcome measures of the gait tasks of the SPAX-app excluding the use of walking aids.

| **Outcome measure** | **SPAX-app** | **SPAX-app** | **p-value*****  Mann-Whitney U | **ICC** |
| --- | --- | --- | --- | --- |
| **Gait** | **HC (n=10)** | **SPAX (n=13)** |  |  |
| Step time (s) | 0.45 ± 0.02 | 0.54 ± 0.05 | **0.0004** | **0.73*** |
| Step time SD (s) | 0.09 ± 0.008 | 0.02 ± 0.01 | **0.0002** | **0.68*** |
| Turn duration (s) | 1.50 ± 0.09 | 2.19 ± 0.50 | **0.00008** | **0.88**** |

** p ≤ 0.05; ** p ≤ 0.01, ***p-values were Bonferroni corrected. A p-value of ≤ 0.0167 was considered statistically significant.
SPAX = spastic ataxias; SD = Standard deviation.*

**Supplemental Table 6:** Cross-sectional validation of the gait task of the SPAX-app with clinical measures in subjects with SPAX excluding the use of walking aids.

| **Outcome measure** | **SARA**  Spearman correlation | **SARAp&g**  Spearman correlation | **SPAX-app**  **Question**  Spearman correlation |
| --- | --- | --- | --- |
| **Gait (n=13)** |  |  |  |
| Step time (s) | 0.45 | **0.82**** | **0.76**** |
| Step time SD (s) | 0.10 | 0.45 | 0.54 |
| Turn duration (s) | 0.25 | 0.42 | 0.55 |

** p ≤ 0.05; ** p ≤ 0.01
SPAX = spastic ataxias; SARA = Scale for Assessment and Rating of Ataxia; SARAp&g = SARA posture and gait score; SD = Standard deviation.*


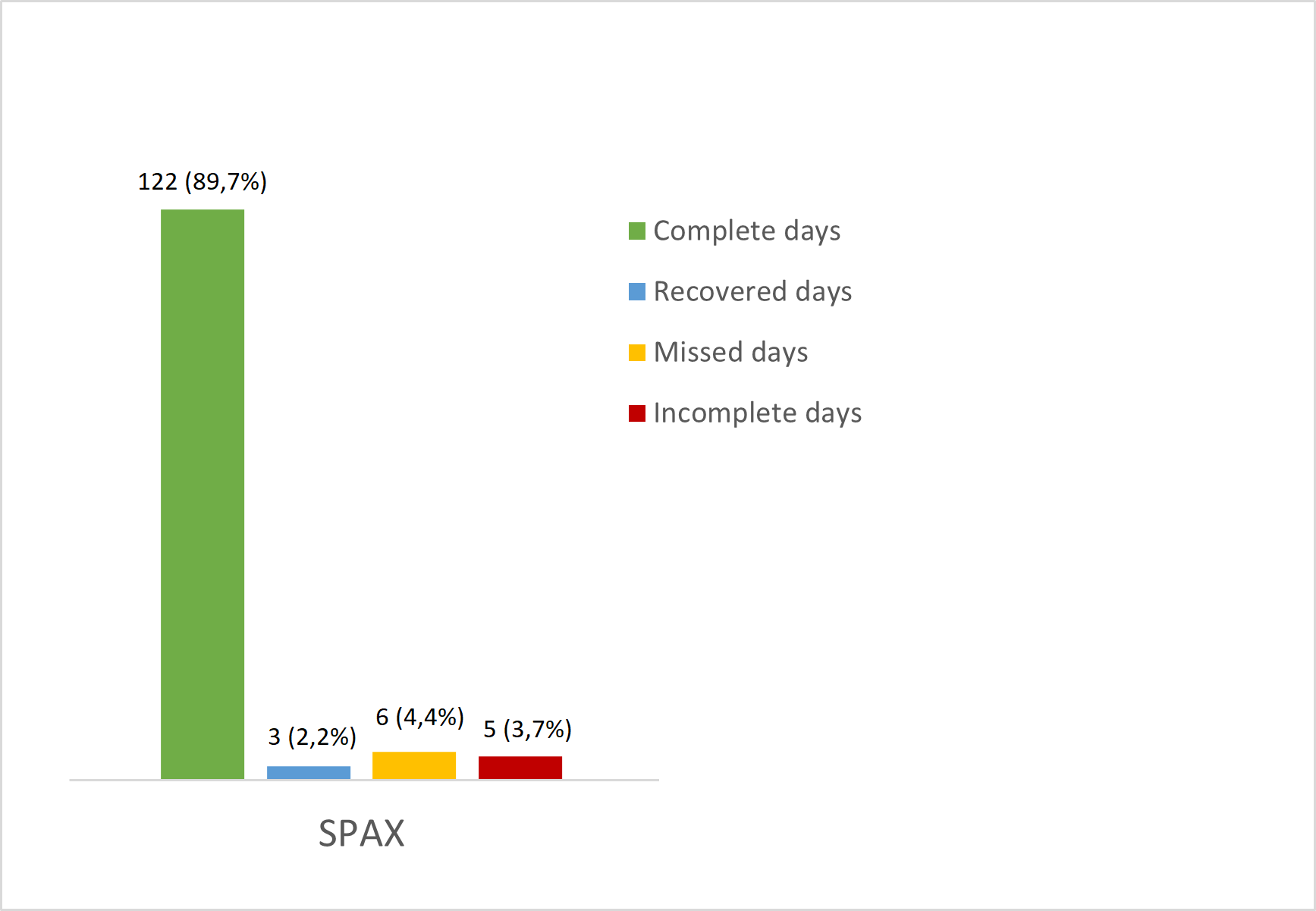


**Supplemental Fig. 2** - The bar graph depicts the number of days that the SPAX-app was used and all tasks were completed (Complete days), the number of the days that the tasks were completed the day after a predetermined day (Recovered days), the number of days that all tasks should have been completed but were not (Missed days) and the number of days that the tasks were incomplete (Incomplete days) for subjects with spastic ataxia (SPAX).

**Supplemental Table 7** – Results of the paired t-test to assess the learning effect for the 4 short motor tasks of the SPAX-app in SPAX subjects.

| **SPAX-app outcome measure** | **SPAX** | | |
| --- | --- | --- | --- |
|  | **Week 1** | **Week 4** | **p-value** |
| **Gait** | n=9 | |  |
| Step time (s) | 0.53 ± 0.05 | 0.53 ± 0.05 | 0.97 |
| Step time SD (s) | 0.14 ± 0.03 | 0.12 ± 0.03 | **0.002** |
| Turn duration (s) | 2.01 ± 0.41 | 2.12 ± 0.38 | 0.11 |
|  |  |  |  |
| **Finger movements** | n=17 | |  |
| Inter-onset interval (s) | 0.44 ± 0.17 | 0.39 ± 0.13 | **0.05** |
| Inter-onset interval SD (s) | 0.11 ± 0.08 | 0.09 ± 0.08 | 0.46 |
|  |  |  |  |
| **Hand movements** | n=17 | |  |
| Duration of hand alternation (s) | 0.95 ± 0.28 | 0.95 ± 0.24 | 0.88 |
| Duration of hand alternation SD (s) | 0.11 ± 0.10 | 0.14 ± 0.13 | 0.49 |

*SPAX = spastic ataxia; SD = Standard deviation.
p-values were not Bonferroni corrected.*

**Supplemental Table 8** – Correlations between lab-based assessments with the SPAX-app and home-based assessments with the SPAX-app.

| **SPAX-app outcome measure** | **SPAX** | | |
| --- | --- | --- | --- |
|  | **Lab** | **Home** | **Spearman correlation** |
| **Gait** | n=8 | |  |
| Step time (s) | 0.55 ± 0.04 | 0.54 ± 0.05 | **0.98**** |
| Step time SD (s) | 0.14 ± 0.03 | 0.14 ± 0.03 | 0.64 |
| Turn duration (s) | 2.07 ± 0.34 | 2.01 ± 0.44 | **0.74*** |
|  |  |  |  |
| **Finger movements** | n=16 | |  |
| Inter-onset interval (s) | 0.41 ± 0.18 | 0.44 ± 0.17 | **0.95**** |
| Inter-onset interval SD (s) | 0.11 ± 0.10 | 0.11 ± 0.09 | **0.56*** |
|  |  |  |  |
| **Hand movements** | n=16 | |  |
| Duration of hand alternation (s) | 0.91 ± 0.25 | 0.94 ± 0.28 | **0.79**** |
| Duration of hand alternation SD (s) | 0.10 ± 0.10 | 0.11 ± 0.11 | 0.40 |

** p ≤ 0.05; ** p ≤ 0.01
SPAX = spastic ataxia; SD = Standard deviation.*

**Supplemental Table 9** – Cross-sectional correlations of outcome measures derived from the APDM wearable sensors during the gait and stance task with clinical measures in subjects with SPAX.

| **APDM Mobility lab**  **outcome measure** | **SARA**  Spearman correlation | **SARAp&g**  Spearman correlation |
| --- | --- | --- |
| **Gait (n=16)** |  |  |
| Step time (s) | **0.68**** | **0.76**** |
| Step time SD (s) | **0.76**** | **0.84**** |
| Turn duration (s) | -0.01 | -0.21 |
|  |  |  |
| **Stance (n=16)** |  |  |
| Sway range (m/s²) | 0.44 | 0.28 |
| RMS Sway (m/s²) | 0.39 | 0.39 |
| Path length (m/s²) | 0.49 | 0.45 |

** p ≤ 0.05; ** p ≤ 0.01
SARA = Scale for Assessment and Rating of Ataxia; SARAp&g = SARA posture and gait score; SD = Standard deviation; RMS = Root mean square.*

**References**

1. Khandelwal SW, N. Identification of Gait Events using Expert Knowledge and Continuous Wavelet Transform Analysis. Proceedings of the International Conference on Bio-inspired Systems and Signal Processing*.* 2014. pp. 197-204.

2. Alafeef M and Fraiwan M. On the diagnosis of idiopathic Parkinson’s disease using continuous wavelet transform complex plot. Journal of Ambient Intelligence and Humanized Computing 2018: 10:2805-15. doi 10.1007/s12652-018-1014-x

3. Palmerini L, Rocchi L, Mellone S, Valzania F and Chiari L. Feature selection for accelerometer-based posture analysis in Parkinson's disease. IEEE Trans Inf Technol Biomed 2011: 15:481-90. doi 10.1109/TITB.2011.2107916

4. Mancini M, Salarian A, Carlson-Kuhta P, Zampieri C, King L, Chiari L and Horak F. Isway a sensitive, valid and reliable measure of postural control. JOURNAL OF NEUROENGINEERING

AND REHABILITATION 2012: 59.

5. Mellone S, Palmerini L, Cappello A and Chiari L. Hilbert-Huang-based tremor removal to assess postural properties from accelerometers. IEEE Trans Biomed Eng 2011: 58:1752-61. doi 10.1109/TBME.2011.2116017
